# Size and shape of the associations of glucose, HbA_1c_, insulin and HOMA-IR with incident type 2 diabetes: the Hoorn Study

**DOI:** 10.1007/s00125-017-4452-7

**Published:** 2017-10-10

**Authors:** Carolien Ruijgrok, Jacqueline M. Dekker, Joline W. Beulens, Ingeborg A. Brouwer, Veerle M. H. Coupé, Martijn W. Heymans, Femke P. C. Sijtsma, David J. Mela, Peter L. Zock, Margreet R. Olthof, Marjan Alssema

**Affiliations:** 1 Department of Epidemiology and Biostatistics, Amsterdam Public Health Research Institute, VU University Medical Center, De Boelelaan 1089a, 1081 HV Amsterdam, the Netherlands; 2 Julius Center for Health Sciences and Primary Care, University Medical Center Utrecht, Utrecht, the Netherlands; 3 Department of Health Sciences, Faculty of Earth & Life Sciences, Vrije Universiteit Amsterdam, Amsterdam Public Health Research Institute, Amsterdam, the Netherlands; 4 Unilever Research and Development, Vlaardingen, the Netherlands

**Keywords:** 2 h post-load glucose, Fasting insulin, Fasting plasma glucose, HbA_1c_, HOMA-IR, Incident type 2 diabetes

## Abstract

**Aims/hypothesis:**

Glycaemic markers and fasting insulin are frequently measured outcomes of intervention studies. To extrapolate accurately the impact of interventions on the risk of diabetes incidence, we investigated the size and shape of the associations of fasting plasma glucose (FPG), 2 h post-load glucose (2hPG), HbA_1c_, fasting insulin and HOMA-IR with incident type 2 diabetes mellitus.

**Methods:**

The study population included 1349 participants aged 50–75 years without diabetes at baseline (1989) from a population-based cohort in Hoorn, the Netherlands. Incident type 2 diabetes was defined by the WHO 2011 criteria or known diabetes at follow-up. Logistic regression models were used to determine the associations of the glycaemic markers, fasting insulin and HOMA-IR with incident type 2 diabetes. Restricted cubic spline logistic regressions were conducted to investigate the shape of the associations.

**Results:**

After a mean follow-up duration of 6.4 (SD 0.5) years, 152 participants developed diabetes (11.3%); the majority were screen detected by high FPG. In multivariate adjusted models, ORs (95% CI) for incident type 2 diabetes for the highest quintile in comparison with the lowest quintile were 9.0 (4.4, 18.5) for FPG, 6.1 (2.9, 12.7) for 2hPG, 3.8 (2.0, 7.2) for HbA_1c_, 1.9 (0.9, 3.6) for fasting insulin and 2.8 (1.4, 5.6) for HOMA-IR. The associations of FPG and HbA_1c_ with incident diabetes were non-linear, rising more steeply at higher values.

**Conclusions/interpretation:**

FPG was most strongly associated with incident diabetes, followed by 2hPG, HbA_1c_, HOMA-IR and fasting insulin. The strong association with FPG is probably because FPG is the most frequent marker for diabetes diagnosis. Non-linearity of associations between glycaemic markers and incident type 2 diabetes should be taken into account when estimating future risk of type 2 diabetes based on glycaemic markers.

## Introduction

Increased levels of glucose and insulin, in the fasting and postprandial state, and HbA_1c_ are key characteristics of type 2 diabetes mellitus. Increments in these risk factors, years before disease onset, precede the development of diabetes [1, 2]. Increases in post-load glucose have been shown to become apparent before increases in fasting glucose [2]. When people reach the impaired glucose tolerance (IGT) state, the transition from IGT to type 2 diabetes within populations occurs at a remarkably high rate: an estimated 25% of people with IGT develop type 2 diabetes within 3–5 years and 40–50% within 10 years [3].

Intervention studies for drugs, lifestyle or diet often use glycaemic markers as their primary outcome measures. To extrapolate the impact of such interventions on diabetes incidence and to estimate their public health impact, accurate quantification of the association between glycaemic markers and incident type 2 diabetes is needed. Knowledge of these associations may subsequently be used to build health impact models that estimate the effect of different interventions on diabetes incidence.

Despite this, only a limited number of studies have reported on the independent continuous associations of fasting plasma glucose (FPG), 2 h post-load glucose (2hPG), HbA_1c_ and fasting insulin with incident type 2 diabetes mellitus. Some studies have investigated the association between impaired fasting glucose (IFG) or IGT status vs normoglycaemia on diabetes incidence [3–6], rather than reporting risks for the entire glucose distribution. Other studies have investigated the continuous association of glycaemic markers with risk of diabetes mostly assuming a linear association [3, 7–10]. To our knowledge, a non-linear shape of the association between glycaemic markers and incident type 2 diabetes has only been previously described for FPG and HbA_1c_, with both showing a curvilinear relationship [11]. The shapes of the associations of 2hPG, fasting insulin and HOMA-IR, a measure of insulin resistance [12], with incident type 2 diabetes have not been extensively studied.

The aim of this study was to assess the size and shape of the associations of FPG, 2hPG, HbA_1c_, fasting insulin, and HOMA-IR with incident type 2 diabetes.

## Methods

### Study population

The Hoorn Study is a population-based prospective cohort study on glucose metabolism performed in the city of Hoorn, the Netherlands. Details of the study have been described previously [13]. In brief, a random sample of 3553 residents from the municipal registry of Hoorn, aged 50–75 years, was invited to participate in the study. In total, 2484 participants were included in the baseline examination from October 1989 to February 1992. Of the original cohort, 150 had died, 108 had moved out of Hoorn and 140 could not be invited for logistical reasons. Of the remaining 2086 individuals who were invited to participate in the follow-up examination from January 1996 to December 1998, 1513 (72.5%) participated [3]. In the present study, we excluded 20 participants with missing data on glycaemic markers at follow-up. We further excluded 144 participants with diabetes at baseline; of whom 41 had known diabetes and 103 were detected at screening (FPG ≥ 7.0 mmol/l or 2hPG ≥ 11.1 mmol/l or HbA_1c_ ≥ 6.5% [48 mmol/mol]). Therefore, 1349 participants were included in the present analyses. The study was approved by the Ethics Committee of the VU University Medical Center and written informed consent was obtained from all participants.

### Baseline measures

Weight (kg) and height (cm) were determined according to standardised procedures in participants wearing light clothes without shoes [14]. BMI was calculated as weight in kilograms divided by height in metres squared (kg/m^2^). Waist circumference (cm) was measured in standing position midway between the lower rib margin and the iliac crest. Systolic blood pressure (mmHg) was measured twice on the right arm with a random-zero sphygmomanometer (Hawksley-Gelman, Lancing, Sussex, UK) while participants were sitting after 5 min of rest, and the mean of the two readings was used for analysis. Information on family history of diabetes, physical activity and smoking were self-reported. Information about alcohol consumption was obtained by a validated semi-quantitative food frequency questionnaire, in which participants were questioned about how many glasses of several alcohol beverages they usually consume per week [15].

For the measurement of FPG levels a venous blood glucose sample was collected after an overnight fast. Subsequently, a standard 75 g OGTT was performed and a blood sample was drawn 2 h after ingestion to measure the 2hPG level. FPG and 2hPG levels were determined by the glucose dehydrogenase method (Merck, Darmstadt, Germany; interassay CV of 1.4%). HbA_1c_ levels were determined in fasting whole blood by ion exchange HPLC (Bio-Rad Diamat, Veenendaal, the Netherlands; interassay CV of 0.6–3.1%). Fasting specific serum insulin levels were quantified by an insulin-specific double-antibody radioimmunoassay (antibody SP21; Linco Research, St Louis, MO, USA; interassay CV of 6% at insulin levels in the range of 40–1000 pmol/l). HOMA-IR was calculated as [fasting glucose (mmol/l) x fasting insulin (pmol/l)]/135 [16]. Participants with elevated levels of glycaemic markers at baseline were advised to take their screening results to their general practitioner.

Triacylglycerol was measured in fasting blood samples by enzymatic techniques (Boehringer-Mannheim, Mannheim, Germany). LDL-cholesterol levels were calculated with the Friedewald formula [17] only for participants with a triacylglycerol level ≤ 4.5 mmol/l because the formula is not reliable above this level.

### Outcome measure: incident type 2 diabetes

At the follow-up examination from January 1996 to December 1998, measures of FPG, 2hPG and HbA_1c_ were determined with the same procedures and assays as previously described for the baseline examination. Incident type 2 diabetes was defined as newly diagnosed diabetes at follow-up. In line with the WHO 2011 diagnostic criteria [18], participants were diagnosed with type 2 diabetes when they met at least one of the following criteria: FPG ≥ 7.0 mmol/l or 2hPG ≥ 11.1 mmol/l or HbA_1c_ ≥ 6.5% (48 mmol/mol) or known diabetes at follow-up (i.e. defined by self-reported current treatment with insulin or hypoglycaemic agents). Medical records were checked in case of doubt to verify diabetes status.

### Statistical analysis

Baseline characteristics of the study participants are summarised by baseline quintiles of FPG. Data are presented as mean (SD) for normally distributed continuous variables or median (25th–75th percentile) for positively skewed distributions. Categorical variables are presented as percentages. Independent variables with positively skewed distributions were transformed taking the natural logarithm (log_*e*_) prior to further analysis. The follow-up duration was calculated as the time between the baseline and follow-up examinations. In order to investigate possible collinearity between independent baseline variables, the correlations between FPG, 2hPG, HbA_1c_, fasting insulin and HOMA-IR were determined using Spearman correlation coefficients.

Multiple imputation techniques were performed to handle missing data in baseline independent variables. As the percentage of missing values was < 5% for all variables, except for physical activity (9%), five imputed datasets were created and the results of the analyses from the different imputed datasets were pooled using Rubin’s rules [19]. The Multivariate Imputation by Chained Equations (MICE) algorithm was used with the predictive mean matching method.

Logistic regression models were used to estimate the ORs for incident diabetes per SD, and per quintile (Q) of FPG, 2hPG, HbA_1c_, fasting insulin and HOMA-IR relative to the lowest quintile. Model 1 was adjusted for age, sex and follow-up duration because participants with elevated glucose levels had shorter follow-up durations. In model 2, additional adjustment was made for potentially confounding factors which were determined a priori. Such a model quantifies, for example, the association of incident diabetes per SD increase in FPG, given that all the other variables in the model stay the same. BMI, waist circumference, systolic blood pressure, triacylglycerol and LDL-cholesterol were all modelled as continuous variables. Smoking status (non-smokers vs smokers), alcohol consumption (0 g/day [reference category], < 10 g/day, 10–30 g/day, ≥ 30 g/day), physical activity (< or ≥ 30 min physical activity per day in summer) and family history of diabetes (parent only with diabetes, sibling only with diabetes or parent and sibling with diabetes) were all modelled as categorical variables. In model 3, the independent variables were mutually adjusted for each other, together with the previously mentioned covariates, in order to examine whether the associations of FPG, 2hPG, HbA_1c_, fasting insulin and HOMA-IR with incident type 2 diabetes were independent of each other. A two-sided *p* value < 0.05 was considered to indicate statistical significance.

Effect-modification was investigated by including interaction terms between baseline blood glucose measures and age (as a continuous variable) and sex, respectively, in the logistic regression models, and calculating their corresponding *p* values. For interaction terms, a *p* value < 0.10 was considered statistically significant.

To investigate the shapes of the associations, logistic regression models including restricted cubic spline (RCS) functions with three knots (percentile 10, 50, 90) were conducted for each independent variable separately [20]. These models were adjusted for age, sex and follow-up duration. The -2 log likelihoods of the logistic regression models with and without RCS functions were compared in the likelihood ratio test (following a χ^2^ distribution with one degree of freedom) to evaluate which model fitted the data best.

In addition, to facilitate comparison of the results of the logistic regression models with RCS functions to the logistic regression models with quintiles of the markers, and to visualise the results, the following was performed. ORs were derived from RCS logistic regression models by calculating the odds at each value of the markers and dividing this by the odds of a reference category. The reference category for calculating the ORs was chosen as the mean value of the first quintile of each marker.

Probabilities for developing type 2 diabetes were computed from the logistic regression models with RCS functions and plotted for each of the markers.

Data analyses were performed using IBM SPSS Statistics version 21 for Windows (SPSS, Chicago, IL, USA) and R version 3.2.3 (www.R-project.org), using the packages rms (https://cran.r-project.org/web/packages/rms/index.html) and ggplot2 (https://cran.r-project.org/web/packages/ggplot2/index.html).

## Results

The mean baseline age of the total study population was 60.3 (6.9) years and 54.6% were male. Relative to participants in the lowest quintile of FPG, those in the highest quintile had higher mean BMI, waist circumference, systolic blood pressure, 2hPG, HbA_1c_, fasting insulin and HOMA-IR (Table 1). Participants in the follow-up examination were on average younger (60.6 years vs 63.2 years), had lower mean baseline FPG levels (5.6 mmol/l vs 5.9 mmol/l), lower 2hPG levels (5.9 mmol/l vs 6.2 mmol/l) and lower HbA_1c_ levels (5.4% [36 mmol/mol] vs 5.6% [38 mmol/mol]) compared with non-participants (*n* = 971).

**Table 1:** Characteristics of the Hoorn Study participants by baseline quintiles of FPG

Characteristic	*n*	Q1 (FPG < 5.0 mmol/l)	Q2 (FPG 5.0–5.3 mmol/l)	Q3 (FPG 5.3–5.5 mmol/l)	Q4 (FPG 5.5–5.9 mmol/l)	Q5 (FPG 5.9–7.0 mmol/l)	Total population
Age (years)	1349	58.9 (6.7)	60.1 (6.9)	59.9 (6.9)	60.9 (6.6)	61.9 (7.0)	60.3 (6.9)
Sex (% male)	1349	33.3	43.3	46.4	52.2	51.9	54.6
FPG (mmol/l)	1349	4.7 (0.2)	5.1 (0.1)	5.3 (0.1)	5.6 (0.1)	6.2 (0.3)	5.4 (0.5)
2hPG (mmol/l)	1349	4.8 (1.3)	5.0 (1.3)	5.4 (1.6)	5.5 (1.6)	6.4 (1.9)	5.4 (1.6)
HbA_1c_ (%)	1348	5.2 (0.4)	5.2 (0.4)	5.3 (0.4)	5.3 (0.5)	5.4 (0.5)	5.3 (0.5)
HbA_1c_ (mmol/mol)	1348	33 (4.4)	33 (4.4)	34 (4.4)	34 (5.5)	36 (5.5)	34 (5.5)
Fasting insulin (pmol/l)	1327	64.4 (52.5–79.5)	71.2 (60.2–86.6)	71.8 (57.0–94.3)	79.3 (62.9–98.8)	89.7 (70.8–111.9)	74.7 (59.9–94.3)
HOMA-IR	1327	2.2 (1.8–2.8)	2.7 (2.3–3.3)	2.8 (2.3–3.8)	3.3 (2.7–4.2)	4.1 (3.2–5.2)	2.9 (2.3–3.9)
BMI (kg/m^2^)	1347	25.2 (2.9)	26.1 (3.0)	26.2 (2.9)	26.6 (3.2)	27.2 (3.3)	26.2 (3.1)
Waist circumference (cm)	1345	84.6 (9.6)	89.0 (9.5)	89.0 (9.7)	91.5 (9.4)	93.8 (10.4)	89.6 (10.2)
Systolic blood pressure (mmHg)	1348	126.6 (18.3)	130.1 (17.1)	130.0 (19.2)	135.1 (19.0)	140.9 (20.5)	132.5 (19.4)
Antihypertensive drug treatment (%)	1349	11.8	12.5	13.8	17.5	21.8	15.4
LDL-cholesterol (mmol/l)	1347	4.5 (1.1)	4.6 (1.0)	4.5 (1.0)	4.6 (1.0)	4.6 (1.0)	4.6 (1.0)
Triacylglycerol (mmol/l)	1349	1.2 (0.9–1.5)	1.2 (1.0–1.7)	1.3 (1.0–1.7)	1.4 (1.0–1.8)	1.5 (1.2–2.1)	1.3 (1.0–1.8)
Family history of diabetes (%)	1346						
Parent only with diabetes		11.5	16.4	9.4	10.2	12.8	12.1
Sibling only with diabetes		2.9	3.5	5.8	5.4	4.1	4.3
Parent and sibling with diabetes		1.8	2.8	1.3	2.5	4.1	2.5
Physical activity (h/day) (%)	1226						
Insufficient (< 0.5 h/day)		8.0	12.4	9.1	9.9	12.9	10.4
Current smoker (%)	1344	31.3	29.5	30.8	28.4	29.5	29.8
Alcohol consumption (%)	1329						
0 g/day		29.7	28.3	25.2	25.6	28.0	27.4
< 10 g/day		44.2	42.8	45.1	42.6	38.1	42.6
10–30 g/day		21.4	23.6	23.4	22.7	24.3	23.0
> 30 g/day		4.7	5.3	6.3	9.1	9.6	7.0

Data are presented as mean (SD), median (25th–75th percentile) or percentage

After a mean follow-up duration of 6.4 (0.5) years, 152 participants developed type 2 diabetes (11.3%). The majority of incident cases had isolated FPG ≥ 7.0 mmol/l (61 participants), followed by isolated 2hPG ≥ 11.1 mmol/l (21 participants) and isolated HbA_1c_ ≥ 6.5% [48 mmol/mol] (21 participants). The remainder had a combination of two criteria (33 participants), while 13 participants had type 2 diabetes based on all three criteria. Three patients with incident diabetes were receiving glucose-lowering medication and had normal glucose and HbA_1c_ levels.

The Spearman correlation coefficients among FPG, 2hPG, HbA_1c_ and fasting insulin ranged between 0.04 and 0.31. As a consequence of high correlations between HOMA-IR and its components (HOMA-IR and FPG: *r* = 0.50; HOMA-IR and fasting insulin: *r* = 0.97), the mutually adjusted model of HOMA-IR was only adjusted for 2hPG and HbA_1c_ in order to avoid possible problems with collinearity.

The associations of FPG, 2hPG, HbA_1c_, fasting insulin and HOMA-IR with type 2 diabetes risk are shown in Table 2. No interactions were observed between baseline glycaemic markers and age or sex (*p* value interaction terms > 0.10). In all models, FPG was most strongly associated with incident diabetes, followed by 2hPG, HbA_1c_, HOMA-IR and fasting insulin. After multivariate adjustment (model 2), all markers, except fasting insulin, were significantly associated with diabetes incidence. Mutual adjustment for other markers (model 3), slightly attenuated the associations.

**Table 2:** Associations of baseline FPG, 2hPG, HbA_1c_, fasting insulin and HOMA-IR with incident type 2 diabetes mellitus

Variable	*n*	Incident diabetes (*n*)	Model 1^a^ OR (95% CI)	Model 2^b^ OR (95% CI)	Model 3^c^ OR (95% CI)
FPG (per SD increase, 0.5 mmol/l)			2.7 (2.2, 3.3)	2.5 (2.0, 3.1)	2.1 (1.7, 2.7)
FPG Q1 (< 5.0 mmol/l)	279	10	1.0	1.0	1.0
FPG Q2 (5.0–5.3 mmol/l)	289	17	1.6 (0.7, 3.6)	1.4 (0.6, 3.1)	1.4 (0.6, 3.2)
FPG Q3 (5.3–5.5 mmol/l)	224	14	1.7 (0.7, 3.9)	1.6 (0.7, 3.6)	1.3 (0.6, 3.1)
FPG Q4 (5.5–5.9 mmol/l)	314	32	2.8 (1.4, 5.9)	2.4 (1.1, 5.1)	1.9 (0.9, 4.1)
FPG Q5 (> 5.9 mmol/l)	243	79	11.2 (5.6, 22.4)	9.0 (4.4, 18.5)	6.0 (2.8, 12.9)
2hPG (per SD increase, 1.6 mmol/l)			2.3 (1.9, 2.8)	2.2 (1.8, 2.6)	1.8 (1.5, 2.3)
2hPG Q1 (< 4.1 mmol/l)	267	10	1.0	1.0	1.0
2hPG Q2 (4.1–4.9 mmol/l)	277	12	1.2 (0.5, 2.8)	1.1 (0.5, 2.6)	1.0 (0.4, 2.4)
2hPG Q3 (4.9–5.7 mmol/l)	280	25	2.5 (1.2, 5.2)	2.0 (0.9, 4.4)	1.8 (0.8, 4.1)
2hPG Q4 (5.7–6.6 mmol/l)	250	32	3.7 (1.8, 7.7)	3.1 (1.4, 6.5)	2.3 (1.1, 5.1)
2hPG Q5 (> 6.6 mmol/l)	275	73	7.8 (3.9, 15.9)	6.1 (2.9, 12.7)	4.1 (1.9, 8.9)
HbA_1c_ (per SD increase, 0.5% [5.5 mmol/mol])			1.7 (1.4, 2.1)	1.6 (1.3, 1.9)	1.3 (1.1, 1.6)
HbA_1c_ Q1 (< 5.0%)	272	15	1.0	1.0	1.0
HbA_1c_ Q2 (5.0–5.2%)	207	24	2.1 (1.1, 4.1)	2.1 (1.1, 4.3)	1.8 (0.9, 3.8)
HbA_1c_ Q3 (5.2–5.5%)	385	25	1.1 (0.6, 2.0)	1.0 (0.5, 2.0)	0.8 (0.4, 1.7)
HbA_1c_ Q4 (5.5–5.7%)	199	20	1.7 (1.2, 2.4)	1.5 (0.7, 3.1)	1.2 (0.5, 2.5)
HbA_1c_ Q5 (> 5.7%)	285	68	4.4 (2.4, 8.0)	3.8 (2.0, 7.2)	2.7 (1.4, 5.2)
Log_*e*_ fasting insulin (per SD increase, 0.4 pmol/l)			1.3 (1.1, 1.6)	1.1 (0.9, 1.3)	1.0 (0.8, 1.2)
Insulin Q1 (< 56.4 pmol/l)	265	17	1.0	1.0	1.0
Insulin Q2 (56.4–68.1 pmol/l)	264	25	1.5 (0.8, 3.1)	1.4 (0.7, 2.9)	1.5 (0.7, 3.4)
Insulin Q3 (68.1–81.1 pmol/l)	267	26	1.6 (0.8, 3.3)	1.5 (0.7, 3.0)	1.3 (0.6, 2.9)
Insulin Q4 (81.1–100.5 pmol/l)	266	35	2.2 (1.2, 4.2)	1.6 (0.8, 3.1)	1.3 (0.6, 2.6)
Insulin Q5 (> 100.5 pmol/l)	265	49	3.0 (1.6, 5.5)	1.9 (0.9, 3.6)	1.3 (0.6, 2.6)
Log_*e*_ HOMA-IR (per SD increase, 0.5)			1.6 (1.3, 1.9)	1.3 (1.1, 1.6)	1.2 (1.0, 1.5)
HOMA-IR Q1 (< 2.1)	271	13	1.0	1.0	1.0
HOMA-IR Q2 (2.1–2.7)	269	22	1.6 (0.8, 3.4)	1.4 (0.6, 2.9)	1.4 (0.6, 3.2)
HOMA-IR Q3 (2.7–3.3)	269	21	1.5 (0.7, 3.1)	1.3 (0.6, 2.7)	1.4 (0.6, 3.1)
HOMA-IR Q4 (3.3–4.1)	270	38	2.8 (1.4, 5.7)	2.1 (1.0, 4.3)	2.2 (1.0, 4.8)
HOMA-IR Q5 (> 4.1)	270	58	4.5 (2.3, 8.6)	2.8 (1.4, 5.6)	2.4 (1.2, 5.1)

Diabetes defined by: FPG ≥ 7.0 mmol/l or 2hPG ≥ 11.1 mmol/l or HbA_1c_ ≥ 6.5% (48 mmol/mol) or by known diabetes at follow-up

^a^Model 1 adjusted for age, sex and follow-up duration

^b^Model 2 additionally adjusted for BMI, LDL-cholesterol, log_*e*_ triacylglycerol, systolic blood pressure, waist circumference, family history of diabetes, physical activity, alcohol consumption and smoking

^c^Model 3 FPG additionally adjusted for 2hPG, HbA_1c_ and fasting insulin; 2hPG additionally adjusted for FPG, HbA_1c_ and fasting insulin; HbA_1c_ additionally adjusted for FPG, 2hPG and fasting insulin; fasting insulin additionally adjusted for FPG, 2hPG and HbA_1c_; and HOMA-IR additionally adjusted for 2hPG and HbA_1c_

**Fig. 1:**
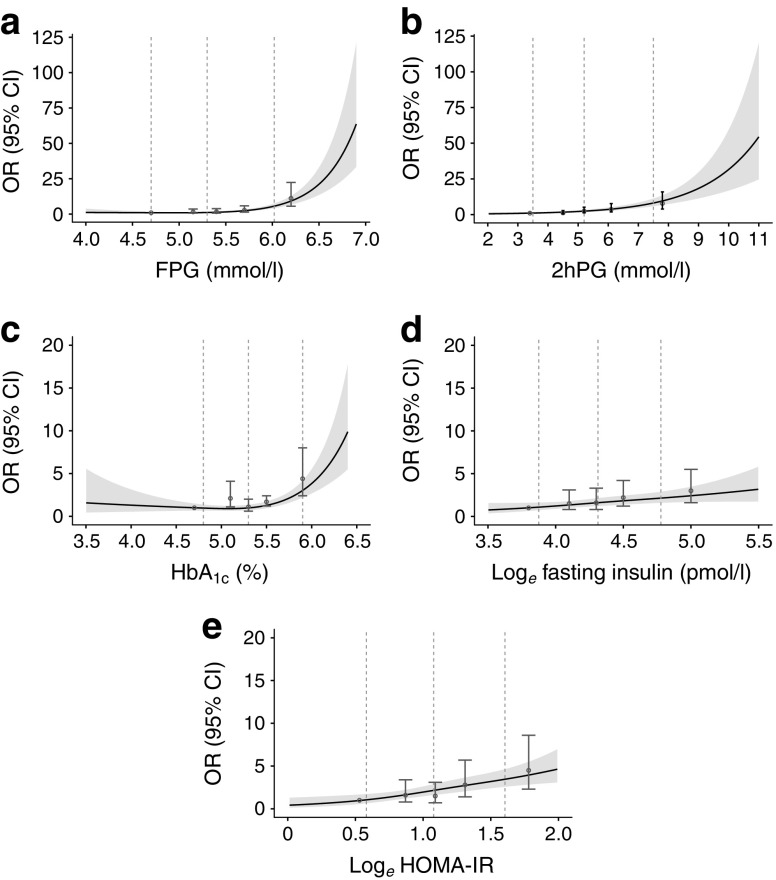
Curves of ORs (95% CI) derived from logistic regression models including restricted cubic splines to evaluate the associations of incident type 2 diabetes with (**a**) FPG (**b**) 2hPG (**c**) HbA_1c_ (**d**) log_*e*_ fasting insulin and (**e**) log_*e*_ HOMA-IR. The dots (95% CI) are the ORs after each risk factor is converted into quintiles with the first dot indicating the lowest quintile, which is used as reference category. Adjustment was made for age, sex, and follow-up duration. The vertical dotted lines indicate the knot locations (percentile 10, 50, 90)

Figure 1 shows the shape of the associations of the glycaemic markers, fasting insulin and HOMA-IR with diabetes incidence. Comparison of the -2 log likelihoods of the logistic regression models with and without RCS functions revealed a statistically significant improvement in model fit for the non-linear RCS models for the glycaemic markers FPG and HbA_1c_ (likelihood ratio tests *p* < 0.001). In contrast, for 2hPG, log_*e*_ fasting insulin and log_*e*_ HOMA-IR (likelihood ratio tests *p* = 0.79, *p* = 0.56 and *p* = 0.28, respectively), the model fit did not significantly improve when including the non-linear RCS functions into the models. Therefore, the shape of the association of FPG and HbA_1c_ with incident type 2 diabetes is non-linear, while the shape of 2hPG, log_*e*_ fasting insulin and log_*e*_ HOMA-IR with incident type 2 diabetes is linear.

**Fig. 2:**
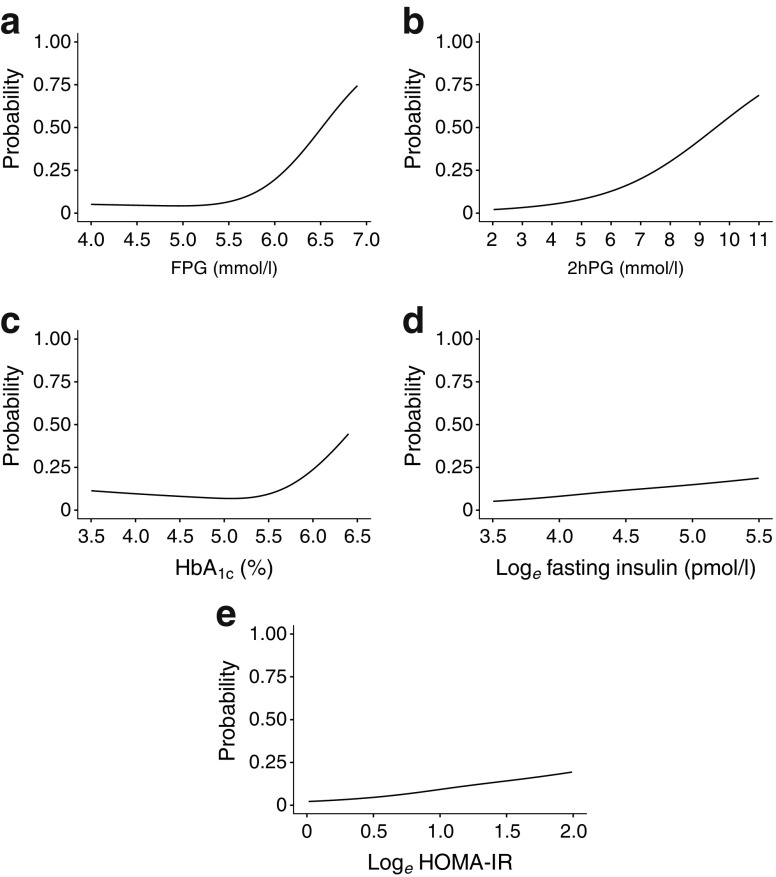
Absolute risks for type 2 diabetes within 6.4 years follow-up for baseline (**a**) FPG (**b**) 2hPG (**c**) HbA_1c_ (**d**) log_*e*_ fasting insulin and (**e**) log_*e*_ HOMA-IR

As shown in Fig. 2, the estimated probability of developing diabetes sharply increases when FPG exceeds 5.6 mmol/l and when HbA_1c_ exceeds 5.5% (37 mmol/mol). For 2hPG, insulin and HOMA-IR, the increment in probability to develop diabetes is more gradual over the whole range of their corresponding distributions.

## Discussion

In this cohort study of a 50–75-year-old Dutch population, FPG was most strongly associated with incident type 2 diabetes followed by 2hPG, HbA_1c_, HOMA-IR and fasting insulin. In multivariate adjusted models, fasting insulin was no longer associated with incident type 2 diabetes. The key finding of this study was non-linearity of the associations of FPG and HbA_1c_ with incident type 2 diabetes, in contrast to 2hPG, log_*e*_ fasting insulin and log_*e*_ HOMA-IR, which were all linearly associated with incident type 2 diabetes.

A limited number of other prospective cohort studies have reported associations for incident type 2 diabetes of all three glycaemic markers, i.e. FPG, 2hPG and HbA_1c_. In the Australian Diabetes, Obesity and Lifestyle Study (AusDiab), a population-based study in which 6537 individuals were followed for over 5 years, FPG (per SD) was the strongest predictor for incident type 2 diabetes, followed by HbA_1c_ and 2hPG, after multivariate adjustment [8]. However, in the KORA S4/F4 cohort study, the association with incident type 2 diabetes was stronger for 2hPG (per SD) than for FPG and HbA_1c_ in multivariate adjusted models [9]. Inconsistencies in results between studies may be explained by the use of different definitions of diabetes. In the AusDiab and KORA studies, type 2 diabetes was defined by FPG and 2hPG criteria, while in our study HbA_1c_ was also included as a diagnostic criterion for incident type 2 diabetes. In addition, the number of people diagnosed with diabetes based on elevated FPG and/or 2hPG levels may differ between cohorts. In our study, the strong association between FPG and incident type 2 diabetes is potentially explained by the high number of individuals who were diagnosed based on FPG. The numbers of participants identified by the individual criteria were not reported for the AusDiab and KORA study.

Another potential explanation for differences in the strengths of associations is diversity in the intra-individual variation between markers. Previous studies have indicated lowest intra-individual variation for FPG and higher intra-individual variations for 2hPG, HbA_1c_ and fasting insulin [21–23]. The higher intra-individual variability of fasting insulin, but also of HbA_1c_, compared with glucose may partly explain the weaker associations with type 2 diabetes [24].

Information on the associations between fasting insulin and HOMA-IR with incident type 2 diabetes is scarce. In the Prospective Study of Pravastatin in the Elderly at Risk (PROSPER) study, HOMA-IR was more strongly associated with incident type 2 diabetes than fasting insulin [25], which corresponds to the observations in our study. Moreover, in the PROSPER study and the San Antonio Heart Study, fasting insulin was significantly associated with the risk of developing type 2 diabetes after adjustment for confounders, while in our study the association between fasting insulin and incident type 2 diabetes was no longer statistically significant after adjusting for metabolic and lifestyle risk factors [25, 26].

In the present study, non-linearity of the associations of FPG and HbA_1c_ with incident type 2 diabetes was indicated by a better model fit of logistic regression models including the RCS functions for these associations. A previous publication showed curvilinear associations for FPG and HbA_1c_ with incident type 2 diabetes [11], which corresponds to the shape of these associations established in our study. Furthermore, the shape of the associations of FPG and HbA_1c_ with incident type 2 diabetes in the present study suggests that the risk plateaus for FPG levels < 5.6 mmol/l and HbA_1c_ levels < 5.5% [37 mmol/mol]. In contrast, the linear associations of 2hPG, log_*e*_ fasting insulin and log_*e*_ HOMA-IR with incident type 2 diabetes suggest that the risk is lowered with lower levels. Contrary to the diagnostic criteria for type 2 diabetes, for which the thresholds are based on associations with complications, thresholds for IFG, IGT and the prediabetes HbA_1c_ are based on associations of the glycaemic markers with incident type 2 diabetes. The presently observed increase in risk from FPG levels about 5.6 mmol/l is closer to the threshold for IFG from the ADA (5.6 mmol/l) [27] than from the WHO (6.1 mmol/l) [18].

Our study thus shows that non-linearity of associations should be taken into account when extrapolating data on risk factors to population risks of type 2 diabetes incidence. For example, in the current study, when a linear association between FPG and incident type 2 diabetes was assumed, a more than twofold increased risk of diabetes for each 0.5 mmol/l increase in FPG was estimated. The current study suggests that at levels < 5.6 mmol/l FPG is not related to diabetes risk, whereas in the higher levels of FPG (> 5.6 mmol/l) the risk is increased two to sixfold. These data are highly relevant for policymakers and public health bodies in future predictions of type 2 diabetes incidence. In addition, this information is relevant when estimating the public health implications of intervention studies targeting glycaemic measures.

The strengths of this study include the use of the 2011 WHO diagnostic criteria, the use of OGTT as well as HbA_1c_ as diagnostic tests for type 2 diabetes mellitus, and the ability to compare the strengths of the associations of incident diabetes with all three glycaemic measures as well as insulin. Our study also has some limitations. First, this study includes a relatively small sample size, which limits the power of the study. Second, screening included only one single blood sample, while in clinical practice two measurements are needed for diabetes diagnosis [28]. Third, the strengths of the associations found in our study were specific to a population aged 50–75 years, which limits the generalisability to broader age ranges. Fourth, participants at the follow-up examination were on average healthier compared with non-participants. Therefore, potential selection bias may have occurred and thus the study population may not be completely representative of the general population. Finally, the fixed follow-up duration of 6 years does not allow the modelling of risks over time.

In conclusion, FPG was most strongly associated with incident diabetes, followed by 2hPG, HbA_1c_, HOMA-IR and fasting insulin. The strong association of FPG compared with 2hPG and HbA_1c_ is in line with FPG being the most common marker for the diagnosis of type 2 diabetes. These results have potential implications for accurately estimating the risk of type 2 diabetes on the basis of glycaemic measures and translating the effects observed in intervention studies into type 2 diabetes risk.

## Abbreviations

2hPG: 2 h post-load glucose
AusDiab: Australian Diabetes, Obesity and Lifestyle Study
FPG: Fasting plasma glucose
IFG: Impaired fasting glucose
IGT: Impaired glucose tolerance
PROSPER: Prospective Study of Pravastatin in the Elderly at Risk
Q: Quintile
RCS: Restricted cubic spline
